# Functional Neurological Symptom Disorder Presenting as Head Titubation: A Rare Case Report

**DOI:** 10.7759/cureus.62248

**Published:** 2024-06-12

**Authors:** Tejas Nehete, Sarang S Raut, Kashish Khurana, Sourya Acharya, Sunil Kumar

**Affiliations:** 1 General Medicine, Jawaharlal Nehru Medical College, Wardha, IND

**Keywords:** functional neurological symptom disorder, cognitive behavioural therapy, dysmenorrhea, chronic depression, titubation

## Abstract

Functional neurological symptom disorder (FND), previously known as conversion disorder, is a condition identified by neurological symptoms that cannot be explained by neurological disease or other medical conditions. FND typically presents with speech disturbances, visual disturbances, paralysis, somatic symptoms like myalgia, and chronic fatigue. This case report describes a case of a 44-year-old female, who presented with dysmenorrhea and had undergone total abdominal hysterectomy with bilateral salpingectomy and manifested as head titubation which was a conversion reaction. On evaluation, it was revealed that the patient had chronic depression and pain. This case report highlights a rare presentation of FND.

## Introduction

The symptoms of functional neurological symptom disorder (FND), previously known as conversion disorder, a mental health condition, impact either motor or sensory function. Despite the lack of an organic basis, FND symptoms have a substantial negative influence on a patient's functioning. Additionally, patients cannot control or feign these symptoms. Sigmund Freud was the first person to use the phrase "conversion disorder" in writing (1856-1939). The psychoanalyst and Austrian neurologist thought that suggesting functional symptoms represents an unconscious conflict [1]. The term "conversion" here means the substitution of a suppressed notion for a physical symptom. Despite evolving understanding, FND remains a complex and poorly understood condition [2].

FND is included in the category of "somatic symptom and related disorders" in the Diagnostic and Statistical Manual of Mental Disorders, Fifth Edition (DSM-5). In previous editions of the DSM, clear psychologic comorbidities were a required part of the diagnosis and the ability to show that the symptoms were not intentionally produced [3]. The diagnostic criterion of "la belle indifférence," or a patient's seeming indifference to their condition, has likewise been eliminated from the DSM-5. These characteristics are now thought to support the FND diagnosis. Dissociative symptoms that are related and recent physical or psychological trauma are additional supporting characteristics [4].

Head titubation is the involuntary rhythmic movements of the head and neck. This can occur continuously or intermittently throughout the day. Differential diagnoses for head tremors include essential tremors, cervical dystonia, Parkinson’s disease, Huntington’s disease, multiple system atrophy (MSA), myoclonus, tardive dyskinesia, and Tourette syndrome [5].

## Case presentation

 A 44-year-old female presented to the hospital with complaints of pain during menstruation (dysmenorrhea) for three months. Additionally, she had a history of prolonged and irregular vaginal bleeding (abnormal uterine bleeding). Medications and the hormonal intrauterine device Mirena, containing 52 mg levonorgestrel, were prescribed three months prior for heavy menstrual bleeding. Despite this, abnormal uterine bleeding persisted even on receiving tranexamic acid and etamsylate. An endometrial biopsy was done which was suggestive of endometrial hyperplasia in the early secretory phase. Hence, the patient was taken for total laparoscopic hysterectomy with bilateral salpingectomy because of persistent abnormal uterine bleeding, not responding to medical treatment and hormonal intrauterine device. The patient was a known case of chronic depressive disorder and was on tab fluoxetine 20 mg once a day, but she was noncompliant and had discontinued it for two months.

On postoperative day 3, the patient went to the washroom and suddenly relatives heard her scream. So, they went inside the washroom and found that the patient was having abrupt onset, continuous head nodding as shown in Video 1. The patient was alert, communicating well, not disoriented, but had severe anxious look on her face.

**Video 1:** Head titubation in the patient and its variability

On general examination, the patient was conscious and oriented to time, place, and person, afebrile to touch, pulse rate was 76 beats per minute, blood pressure was 120/80 mm Hg, blood oxygen saturation was 98% while breathing on ambient air, and respiratory rate was 20 cycles per minute without involvement of accessory muscles of respiration. There was a characteristic head titubation present which was involuntary, fluctuating in amplitude and frequency and intermittently in a non-patterned manner especially reducing during conversation as shown in Video 1. The frequency of head nodding was also reduced while doing her neurological examination. When asked to write her name, titubation reduced further.

The neurological examination produced normal results, encompassing assessments of motor function, sensory perception, and cranial nerve function, along with reflexes and the absence of Babinski sign. No aphasia, dysarthria, or focal neurological deficits were detected during the evaluation. Her labs were within normal limits, as presented in Table 1. 

**Table 1:** Laboratory blood investigations of the patient Notable findings include low hemoglobin (9.9 gm/dL) indicating anemia, which could be related to chronic blood loss and her chronic depressive disorder. All other parameters are within normal limits, supporting the diagnosis of functional neurological symptom disorder without underlying metabolic causes

Laboratory parameters	Laboratory results	Reference range
Haemoglobin	9.9 gm/dL	12-15 gm/dL
Mean corpuscular volume	76.8 fL	83-101 fL
White blood cells	9300 cells/cu mm	4000-10000 cells/cu mm
Platelets	4.03 lakh cells/cu mm	1.50-4.10 lakh cells/cu mm
Aspartate transaminase	27 U/L	14-36 U/L
Alanine transaminase	9 U/L	<35 U/L
Alkaline phosphatase	52 U/L	38-126 U/L
Direct bilirubin	0.2 mg/dL	0.0-0.3 mg/dL
Indirect bilirubin	0.3 mg/dL	0.0-1.1 mg/dL
Total bilirubin	0.5 mg/dL	0.2-1.3 mg/dL
Total protein	7.2 gm/dL	6.3-8.2 gm/dL
Serum albumin	4.0 gm/dL	3.5-5.0 gm/dL
Urea	25 mg/dL	15-36 mg/dL
Serum creatinine	0.9 mg/dL	0.52-1.04 mg/dL
Sodium	144 mmol/L	137-145 mmol/L
Potassium	4.7 mmol/L	3.5-5.1 mmol/L
Calcium	9.1 mg/dL	8.4-10.2 mg/dL
Magnesium	2.0 mg/dL	1.6-2.3 mg/dL
Phosphorus	2.7 mg/dL	2.5-4.5 mg/dL
Random blood sugar	83 mg%	70-150 mg%

**Figure 1:**
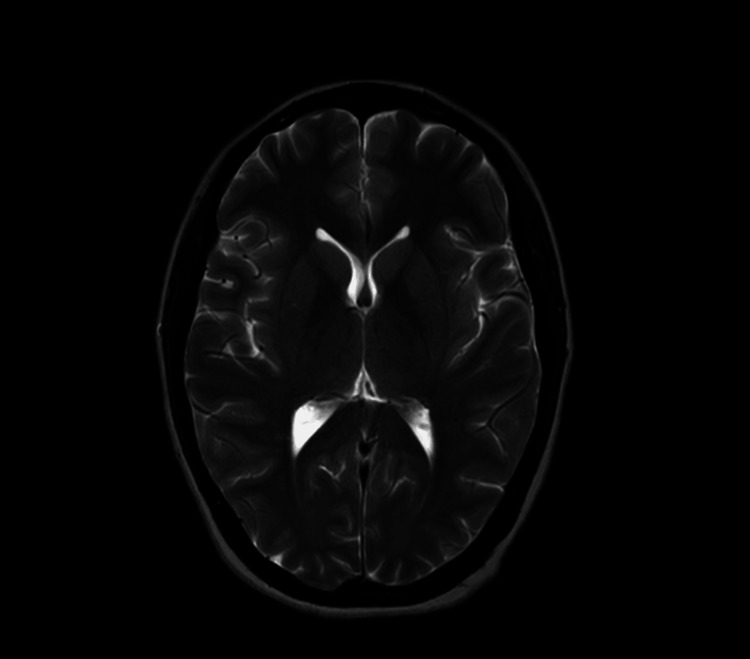
Magnetic resonance imaging (MRI) brain of the patient showing no brain parenchymal abnormalities, supporting the diagnosis of functional neurological symptom disorder (FND) with no underlying organic cause

The patient's magnetic resonance imaging (MRI) of the brain was done, and results indicated no abnormal findings as shown in Figure 1. An electroencephalogram was conducted to rule out seizures, and the results were also unremarkable.

A probable diagnosis of FND was made, and a psychiatric evaluation of the patient was done. Psychiatric history revealed that the patient had chronic depression and anxiety disorder probably due to chronic dysmenorrhea and pain for two to three years and psychotherapy in the form of cognitive behavioral therapy was advised. After three days, her head nodding was significantly relieved as shown in Video 2. The patient was then discharged with advice to continue tab venlafaxine 25 mg three times a day and to follow up after 1 month.

**Video 2:** A significantly relieved head titubation after cognitive behavioral therapy

## Discussion

FND can be precipitated by psychological, social, and biological factors. Many patients with conversion disorder are found to have a history of childhood abuse. FND is usually triggered by depression, trauma (physical and psychological), and acute neurological illness. Typical presentations include speech and visual disturbances, paralysis, and somatic symptoms such as myalgia and chronic fatigue. Typically, abnormal movements in conversion disorders are sudden in onset, fluctuate in frequency and amplitude, are non-patterned, and improve on distraction like in our case [4].

FND, previously known as conversion disorder, is characterized by neurological symptoms unexplained by neurological disease or other medical conditions. Based on presentation and investigations, this patient was diagnosed to have functional tremors. Head titubation is rare in conversion disorder. In this patient, the amplitude and frequency of head nodding changed during various scenarios such as while talking, while getting examined, and when the patient was told to write something on paper. Psychotherapy and cognitive behavioral therapy are the cornerstones [6,7]. For motor-related conversion reactions, physical therapy is also essential. If there exist associated comorbidities like depression and anxiety, then antidepressants and anxiolytics are usually added to the therapeutic regimen [8]. The most commonly used drugs are serotonin and norepinephrine reuptake inhibitors. Atypical antipsychotics like quetiapine may be used. Further research studies also suggest that trans-magnetic stimulation may be beneficial in neurological disorders. The disorder, in this case, was completely relieved after cognitive behavioral therapy. 

## Conclusions

This case highlights a rare clinical presentation of FND. The management of the patient included cognitive behavioral therapy and functional restoration interventions for chronic pain. The important thing to remember is FNDs can be treated after prompt diagnosis with proper counseling and psychiatric therapy.
